# Large language models in patient education for brain tumors: opportunities, risks, and ethical considerations

**DOI:** 10.3389/fonc.2026.1795441

**Published:** 2026-03-24

**Authors:** Rafail C. Christodoulou, Platon S. Papageorgiou, Ana Carolina Lucio Pereira, Elena E. Solomou, Sokratis G. Papageorgiou, Evros Vassiliou, Michalis F. Georgiou

**Affiliations:** 1 Division of Neuroimaging and Neurointervention, Department of Radiology, Stanford University, Stanford, CA, United States; 2 Department of Medicine, Medical School, National and Kapodistrian University of Athens, Athens, Greece; 3 Oncological Research Institute (IPON)/Discipline of Gynecology and Obstetrics, Federal University of Triângulo Mineiro, Uberaba, Brazil; 4 Internal Medicine-Hematology, University of Patras Medical School, Rion, Greece; 5 1st Department of Neurology, Medical School, National and Kapodistrian University of Athens, Eginition Hospital, Athens, Greece; 6 Department of Biological Sciences, Kean University, Union, NJ, United States; 7 Department of Radiology, University of Miami, Miami, FL, United States

**Keywords:** artificial intelligence in healthcare, brain tumors, large language models, neuro-oncology, patient education

## Abstract

**Background:**

Patients with brain tumors often struggle to understand their condition because of complex imaging findings, multidisciplinary care pathways, and frequent cognitive and emotional vulnerability. Effective patient education is, therefore, essential but difficult to deliver within routine clinical encounters.

**Objective:**

This narrative review evaluates the role of large language models (LLMs) in supporting patient education for individuals with brain tumors.

**Content:**

We synthesize evidence from neuro-oncology, radiology, and digital health literature on the use of LLMs to explain imaging results, diagnoses, and treatment options in patient-centered language. Potential benefits include improved health literacy, accessibility, and continuity of education. Key limitations are also discussed, including hallucinations, output variability, overtrust, data privacy concerns, and ethical challenges. A clinician-guided framework for responsible integration is proposed.

**Conclusion:**

When used under clinician supervision as educational support tools, LLMs may enhance patient understanding and engagement in brain tumor care. Safe implementation will require structured governance, oversight, and alignment with ethical standards.

## Graphical Abstract

**Figure:**
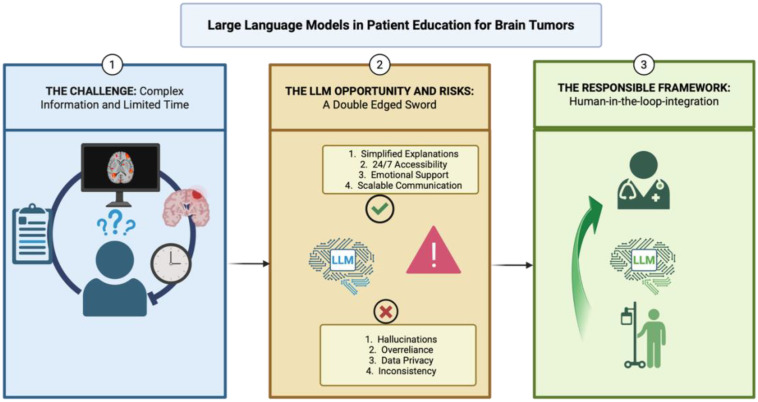

## 1 Introduction

Brain tumors such as gliomas and metastases impose significant clinical and emotional challenges. They frequently occur suddenly with symptoms like seizures or cognitive impairments, leading to neurological problems like memory loss, paralysis, or personality shifts that interfere with daily functioning (1). Unlike many other cancers, a brain tumor directly endangers the mind and personal identity, and caregivers often see their loved ones experience personality shifts and functional changes, truly making it a family disease in every sense (2). Additionally, the prognosis for brain tumors is unfavorable, with malignant tumors like glioblastoma having a median five-year survival rate of below 10% (3). As expected, patients with aggressive tumors experience high distress, with more than one-third of high-grade glioma patients reporting clinically significant distress, which is linked to lower quality of life and an increased need for support (4). This convergence of a sudden life-changing illness, neurological disability, and an exposed outlook imposes significant psychosocial stress on everyone involved.

Providing information and education in this neuro-oncologic context is particularly challenging. Brain tumors include more than 120 different subtypes, each with complex names and treatment plans (5, 6). Patients and families need to understand a wide range of concepts, from surgical risks to chemotherapy, radiation, and new therapies, and all while coping with the shock of diagnosis and often their loved one’s cognitive impairments. Significant health literacy challenges exist, as much of the educational material about brain tumors is written well above the average reading level. For instance, a recent study revealed that none of the patient materials on brain tumor prognosis, whether online or generated by ChatGPT-4, met the recommended readability standards (7). In that particular evaluation, most websites required at least a high school or college reading level, and 93% of ChatGPT’s responses matched a graduate-level reading level, which could impede patients’ capacity to make informed choices. This underscores the communication gap as the information might not be in an accessible format for those who need it (7).

Current methods of patient education in neuro-oncology frequently fail to bridge these gaps. Clinicians effectively explain diagnoses and treatments during visits, but the large amount of information can be overwhelming. Additionally, limited time due to a physician shortage means each patient has only a short time to express their concerns (8). Patients, often anxious and cognitively overloaded, may forget or misunderstand important details. Additionally, surveys in oncology have shown high dissatisfaction among patients and their families regarding the information given following a cancer diagnosis (2). Neuro-oncology patients often face urgent information needs that change as their condition develops. However, these questions are not always fully answered in a single consultation (4, 9). Therefore, families often seek answers online or through support groups, indicating that current communication methods fall short. A qualitative study of brain tumor caregivers found that they felt unprepared and that doctors often couldn’t address all their questions due to limited time (10). These findings highlight that even expert healthcare teams struggle to communicate complex neuro-oncologic information in an understandable way. This gap in care means patients with potentially terminal illnesses might not fully grasp their condition or options, increasing anxiety and reducing trust. Since these patients may experience emotional distress and PTSD symptoms at baseline (11), it is essential for physicians to minimize any additional emotional burden that could result from misinterpreting complex medical knowledge.

Due to many challenges in providing understandable information to patients, solutions using large language models (LLMs) have been explored. Models, like ChatGPT-4, are artificial intelligence (AI) systems trained on vast datasets to generate human-like text. They offer opportunities for patient education by answering questions, tailoring explanations to individual understanding, and providing clarification, tasks that are often limited by clinicians’ time (12). A major benefit is scalability; for instance, while a doctor can see only a limited number of patients, an AI chatbot can interact with many patients simultaneously throughout the day (13).

Early evidence suggests LLMs can communicate empathetically. One study found that AI chatbot responses to patients’ questions were nearly 10 times more likely to be rated as empathetic than those from physicians. Researchers believe chatbots maintain a patient-centered tone by responding to emotional cues with polite, reassuring language, unaffected by fatigue or time pressure, unlike humans (14). In neuro-oncology, where conversations involve fear, hope, and uncertainty, this ability to provide emotional support at scale could be revolutionary. A virtual navigator powered by LLMs could provide ongoing explanations of MRI results, treatment effects, and care, making information more accessible and personalized. This could supplement limited face-to-face time, making patients feel more informed, heard, and supported (15).

Integrating LLMs into patient education presents significant risks and ethical challenges. These models don’t truly understand medicine; they generate responses based on training data, which can lead to inaccuracies or AI hallucinations (16). In cancer care, a confident but wrong answer about treatment or prognosis could cause harm. Concerns include bias, trustworthiness, and patient privacy, as data protection is crucial if patients share personal details. While AI can mimic empathy, it lacks genuine human insight and accountability, risking depersonalized care (17). Regulators advocate careful oversight, transparency, and human verification. Therefore, balancing innovation with safety is vital in neuro-oncology (7).

This review synthesizes current literature on LLMs in neuro-oncology, highlighting opportunities and risks. While brain tumors are a broad category in neuro-oncology that includes diverse disease subtypes with unique imaging, treatment, prognosis, and education needs, such as glioblastoma, meningioma, pituitary adenoma, and metastases. LLM performance and educational utility vary across these. The review explores how LLMs, such as ChatGPT-4, could improve communication about different brain tumor subtypes and address ethical and practical challenges. By combining insights from oncology, communication, and digital health, it guides responsible AI use to empower patients, avoid pitfalls, and support families in neuro-oncology.

## 2 Literature search strategy

A structured literature search was conducted to identify relevant studies examining the role of LLMs in patient education for healthcare, oncology, and neuro-oncology. The search strategy was developed in accordance with established recommendations for transparent reporting in narrative reviews, including the SANRA guidelines (18).

Targeted searches were performed in PubMed/MEDLINE, Embase, and Scopus from January 2008 to January 2026. The starting year was selected to capture foundational literature on patient communication in neuro-oncology while ensuring inclusion of contemporary studies on transformer-based LLMs after 2020. Combinations of controlled vocabulary and keyword terms were used, including variations of: *“large language model,” “ChatGPT,” “GPT-4,” “artificial intelligence,” “patient education,” “health literacy,” “oncology communication,” “brain tumor,” “glioma,”,” brain metastasis” “meningioma,”“ pediatric brain tumors”,” hemangioblastoma”,” vestibular schannoma” and “neuro-oncology.”*.

Studies were included if they:

- Evaluated LLMs or AI-based conversational systems in healthcare contexts.
- Addressed oncology or neuro-oncology communication.
- Examined patient education, readability, empathy, clinical workflow integration, or ethical considerations.
- Were peer-reviewed original studies, systematic reviews, scoping reviews, or substantive policy/ethics analyses.

Studies were excluded if they:

- Focused solely on technical AI architecture without a healthcare application.
- Did not involve clinical, educational, or communication relevance.
- Lacked methodological or analytical contribution.

Titles and abstracts were screened for relevance, followed by full-text review when appropriate. The reference list of key articles was also manually examined to identify additional relevant publications. Given the rapid evolution of LLM research, supplementary references were incorporated to address emerging clinical, ethical, and implementation themes not yet captured in earlier reviews.

This approach emphasizes broad conceptual coverage and clinical relevance rather than exhaustiveness, which may lead to selection bias—a limitation that has been acknowledged. While narrative review methodology does not mandate fully systematic or reproducible search processes, it highlights transparency in the identification and selection of literature. Therefore, a structured database search served as the basis for this synthesis, complemented by targeted citation tracking and the inclusion of recent high-impact studies, guided by expert input (19). Overall, this review includes 63 studies.

## 3 Discussion

### 3.1 Brain tumors and patients’ communication needs

Brain tumors present complex communication challenges because of their biological diversity, reliance on imaging for diagnosis, and effects on cognition and emotions. They directly affect neurological functions such as memory, language, and insight, often when patients already have reduced cognitive capacity. Therefore, consistent, clear, and accessible education is essential throughout the course of the disease.

Neuro-oncology depends on complex imaging, molecular pathology, and multidisciplinary decisions, which are hard for non-medical people to interpret. Studies show that AI systems, including GPT-4 and GPT-4o, perform poorly at diagnosing brain tumors from MRI images, with GPT-4 at 40% and GPT-4o at 70%, compared with 92% for clinicians (20). Notably, when assigned to interpret subtle MRI signs of glioblastoma, most models failed, with only one identifying the high-grade tumor correctly; others suggested diagnoses like demyelination or edema (21). These findings show that neuro-oncologic imaging remains complex and often unclear, even for advanced AI systems. Radiologist reports can be intricate, making interpretation difficult for patients with fewer resources. The challenge extends beyond diagnosis; treatment planning for brain tumors requires careful consideration of tumor grade, functional status, surgical scope, and additional therapies. When ChatGPT was tested for decision-making in glioma adjuvant therapy, experts showed low agreement on diagnosis but moderate-to-high consensus on treatment plans and regimen choices (22). While the assessment was conducted on simulated data, it may be a first step toward further verification of LLMs’ ability to summarize or explain established treatment pathways under clinician supervision. A common theme in neuro-oncology is the gap between what clinicians communicate and what patients actually understand. Neuroimaging plays a key role in diagnosis and follow-up, but there are few tools to help patients interpret these findings meaningfully. An early informatics study showed that a patient-focused radiology portal, which combines imaging timelines with simplified report narratives, was highly supported by clinicians as a way to improve patient understanding (23). However, concerns about potential misinterpretation and unintended disclosures highlighted the challenge of balancing transparency with comprehension. More recent research indicates that patients often seek external explanations when official education is lacking. In places where delays in seeking care for brain tumor symptoms are common, ChatGPT was tested as a simulated advisor for symptom interpretation. The model reliably advised hospital visits for simulated brain tumor cases and encouraged care even when patients hesitated (24). This highlights the need for patients to have accessible, ongoing guidance that reinforces medical advice outside the clinical setting.

**Figure 1:**
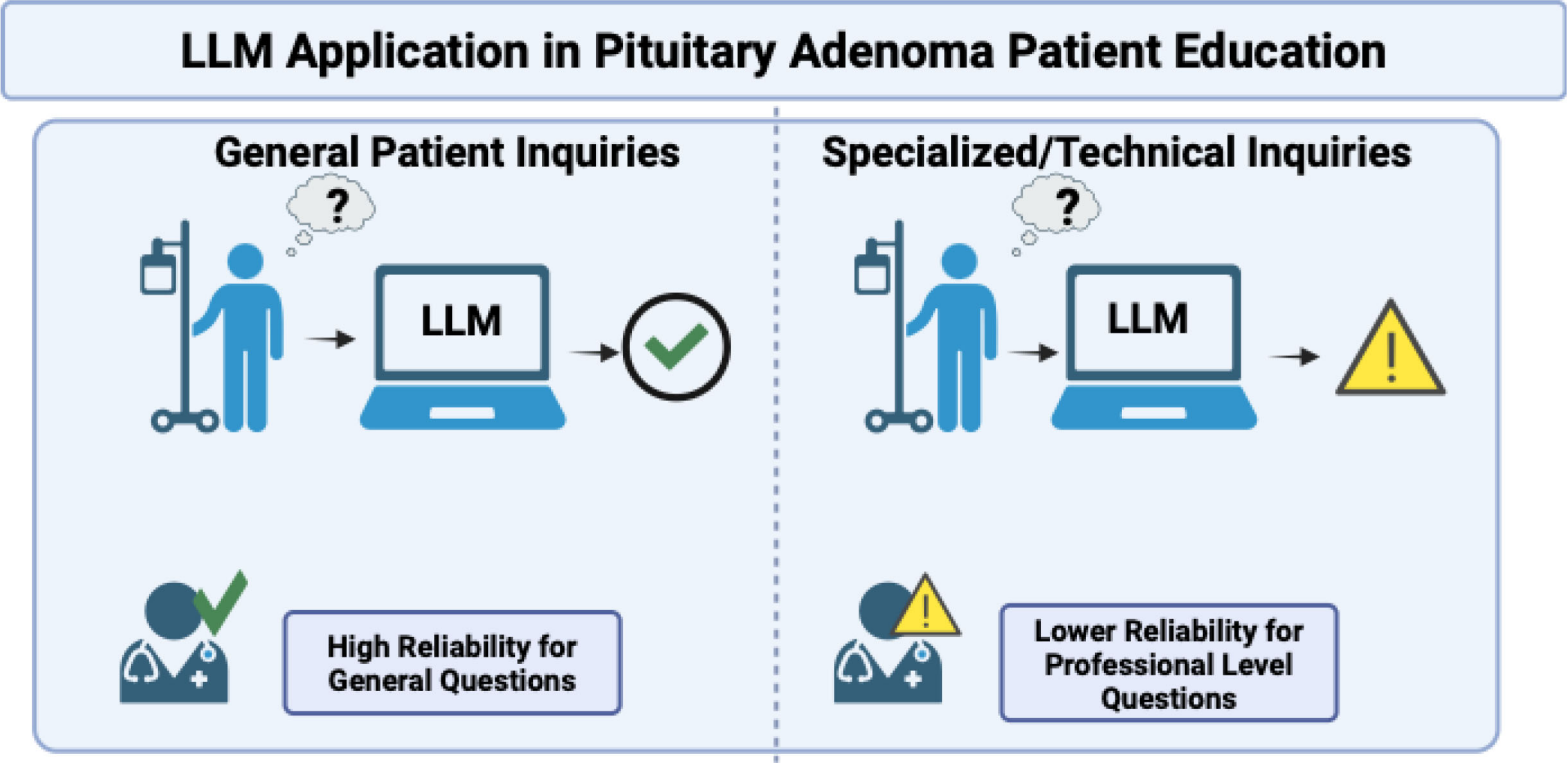
Large language models show high reliability for general patient questions but lower reliability for specialized, professional-level inquiries, supporting their role as clinician-supervised educational tools rather than standalone clinical resources. Created in BioRender. Christodoulou, R. (2026) https://BioRender.com/kaabugv.

**Figure 2:**
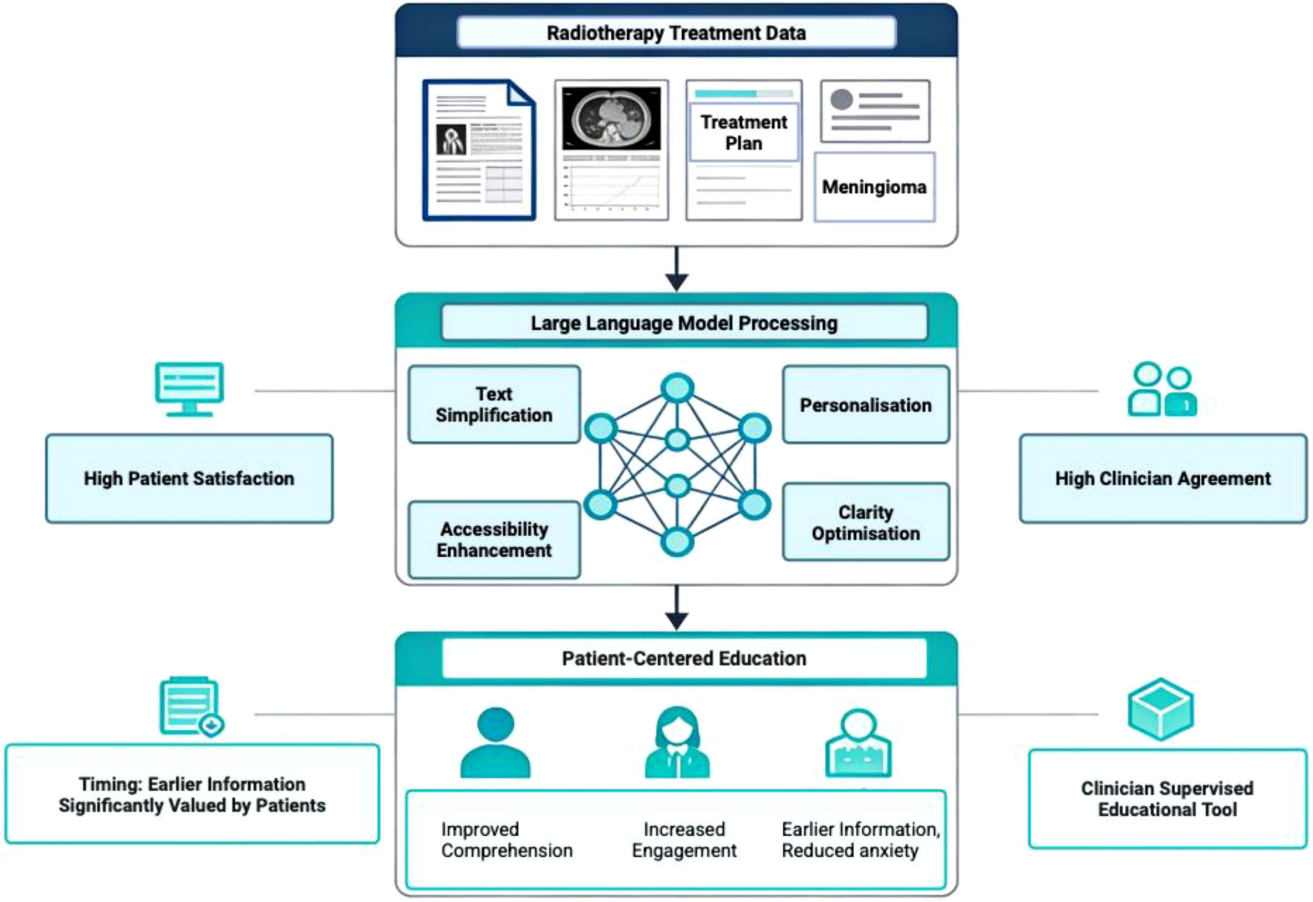
Large language model–assisted patient education framework in neuro-oncology. Large language models transform radiotherapy and imaging data into simplified, personalized, and accessible patient-centered educational content under clinician supervision, improving comprehension, engagement, and satisfaction while maintaining clinical agreement. Created in BioRender. Christodoulou, R. (2026) https://BioRender.com/c924r0c.

Patient education needs vary across brain tumor subtypes, and current evidence remains subtype-specific. For example, in pituitary adenomas, ChatGPT proved highly reliable in answering general patient questions, with over 82% of responses rated as trustworthy by neurosurgeons. It’s important to note that pituitary adenomas differ in biology, prognosis, and treatment, and thus patients’ questions are distinct from those of gliomas, metastases, or meningiomas, so these findings cannot be generalized to other tumor subtypes. However, its performance dropped for more specialized, professional-level queries (**Figure 1**) (25). We must emphasize that this distinction is important because patients mainly seek understanding about their diagnosis, treatment options, and expected outcomes, rather than technical details. A similar trend was observed in meningioma cases, where ChatGPT-generated educational content was rated as clear and relevant by over 90% of patients after radiotherapy, with clinicians largely agreeing on its accuracy (26, 27). Importantly, patients indicated that having this information earlier in their treatment would have been especially helpful, highlighting the importance of timing in patient education (**Figure 2**).

Overall, these findings indicate that brain tumor patients prefer clear explanations providing context about their disease, treatments, and imaging results over decision-making tools they can use independently. The willingness of 60–65% of patients to utilize LLM for future medical inquiries demonstrates openness to digital resources and highlights a gap in current educational materials.

Despite the promise of AI-supported education, multiple studies highlight the risks associated with unsupervised information sharing. LLMs vary in their completeness and accuracy, especially regarding treatment-specific details and side effects (26–28). For instance, errors in tumor localization, grading, and differential diagnosis were prevalent in imaging interpretation tasks, despite moderate-to-high overall acceptability ratings (29). These inconsistencies are particularly problematic in neuro-oncology due to high-stakes information, where misinterpretation can increase patient anxiety or provide false reassurance.

In general, neurooncology patients encounter complex information during vulnerable times, and existing education methods often fall short. LLMs could assist by converting imaging and treatment details into understandable language, complementing clinician guidance, and providing support outside of appointments. Nonetheless, due to inconsistent diagnostic accuracy, these tools should be regarded as educational resources rather than decision-making authorities.

### 3.2 LLMs: a technical weapon for clinicians

LLMs can generate human-like text by learning statistical patterns from large amounts of data. Instead of relying on explicit rules or medical knowledge bases, they use deep neural networks to model relationships between words, phrases, and concepts across billions of examples (30, 31). This allows the network to produce fluent, context-aware responses, though its outputs can seem confident even when inaccurate (32).

LLMs predict the most likely next word given context, trained on diverse datasets, including language, scientific literature, and medical texts. They can summarize, explain, and rephrase complex information, but do not truly understand disease mechanisms or clinical intent (33, 34). Instead, they recognize linguistic patterns linked to medical reasoning, which explains both their strengths and limitations. Recent applications of LLMs have demonstrated increasing capacity to process structured clinical language (35). Studies evaluating their performance on real-world radiology reports show that LLMs can extract salient diagnostic cues from narrative imaging descriptions and generate coherent differential diagnoses (36). LLMs excel when information is pre-processed into text by experts because they benefit from clear, standardized language in reports, thereby minimizing ambiguity (37). However, with raw visual data or poorly structured inputs, performance drops, underscoring their reliance on textual abstraction rather than direct clinical reasoning (38).

Beyond diagnostic summarization, LLMs help translate complex clinical assessments into accessible language. Neuropsychological testing, crucial for preoperative brain tumor evaluation, is often hard for patients to understand. Comparisons of various LLMs show that they can produce clear, patient-friendly explanations of test purposes and results with moderate-to-high accuracy (39). This ability arises from training in explanatory medical texts and educational materials, allowing individuals to reframe technical terminology into simpler narratives while maintaining the original information structure (40).

However, as previously stated, readability is not an inherent strength of LLMs. Analyses of AI-generated neuro-oncology educational content consistently show that default outputs surpass recommended health literacy thresholds, often reaching undergraduate or graduate reading levels (7), indicating a technical bias toward producing linguistically dense, information-rich text unless explicitly guided. Therefore, prompt structure is crucial in influencing output quality. Some experts note that zero-shot prompting, a technique in which no examples or constraints are provided, limits adaptability, whereas structured prompts that request simplified explanations can greatly enhance accessibility (41). Clinicians need to understand that LLM behavior is not fixed but highly sensitive to task framing.

Recent advances have broadened LLM capabilities beyond text processing. Multimodal models now combine visual and textual inputs, allowing for simultaneous interpretation of descriptions and imaging data. Although these systems demonstrate potential for oncology decision support, such as in molecular tumor diagnosis boards, their internal reasoning remains difficult to interpret (42). Variability and occasional dependence on less robust evidence suggest that these systems favor comprehensiveness and fluency over strict clinical reasoning. This stochastic nature stems from their probabilistic design and should be kept in mind when applying them clinically.

LLMs primarily serve as pattern-recognition tools focused on language rather than as autonomous agents. They excel at synthesizing, rephrasing, and contextualizing medical information, particularly when guided by experts (43). However, they have inherent limitations, such as overconfidence and variability, stemming from training on diverse datasets (44). Recognizing these traits helps clinicians interpret outputs accurately and view their role as supportive in patient education rather than as diagnostic tools. This foundational understanding is essential for exploring their application in neuro-oncology patient education.

### 3.3 Applications of large language models in brain tumor patient education

LLMs have been increasingly explored as supportive tools for patient education in neuro-oncology, with most applications focusing on information clarification (45). Across oncology education, LLMs have been evaluated for their ability to explain diagnoses, imaging findings, treatment options, and supportive care considerations in language accessible to non-medical audiences (46). For example, a notable use case is converting complex diagnostic data into explanations that patients can understand. In neuropsychological assessments, LLMs have shown the ability to clearly explain the test purpose, methodology, and results, with high accuracy as rated by experts (39). Notably, these explanatory capabilities could be especially useful for patients with brain tumors undergoing preoperative cognitive assessments, where results are crucial for surgical planning but are often hard to explain in brief visits. Supporting this idea, a cross-sectional study in radiation oncology with 115 patients’ questions showed that responses generated by LLMs were rated as equal or better than professional society materials in 94% of cases for accuracy, 77% for completeness, and 91% for conciseness. Nonetheless, the readability of these responses exceeded the recommended patient education levels, indicating that high informational quality alone does not guarantee ease of understanding (47). Additionally, LLMs can process imaging information when presented in structured textual form. When provided with radiologist-authored MRI reports rather than raw imaging data, ChatGPT and GPT-4 demonstrated comparable performance in listing differential diagnoses and summarizing probable tumor types within predefined report templates (36, 36). Importantly, these studies evaluated LLM performance using radiologist-authored MRI reports rather than raw imaging data. However, this does not reflect independent image interpretation or diagnostic equivalence to neuroradiologists, as prior studies have shown markedly reduced performance when models are tasked with analyzing subtle MRI findings directly. Although these studies primarily assessed diagnostic agreement within report templates, they also suggest that structured radiology content can be transformed into clearer explanations. Therefore, LLMs may support patient understanding of imaging impressions without requiring access to raw images or highly technical radiology reports. In addition, current clinical workflows often lack scalable communication tools between appointments. In simulated brain tumor consultation scenarios, ChatGPT appropriately emphasized urgency and recommended hospital evaluation when clinically concerning symptoms were described (48), revealing the potential of LLMs to strengthen clinician guidance between appointments, especially in areas with limited specialist access.

Comparative analyses of educational materials show that, although LLMs can produce detailed explanations, their default outputs often exceed the recommended readability levels for patient education (49). Further studies indicate that structured prompting methods can significantly enhance clarity and accessibility, emphasizing that their success depends not only on the model’s capabilities but also on guided implementation. The principal educational applications of LLMs in neuro-oncology, spanning from neuroimaging interpretation to neuropsychological assessment, treatment explanation, and health literacy support, are summarized in **Table 1**.

**Table 1:** Applications of large language models in brain tumor patient education.

Domain	Use case	LLM role	Reference
Neuroimaging communication	MRI diagnosis and follow-up	Simplifies radiology report language into patient-friendly explanations	(36)
Neuropsychological assessment	Preoperative cognitive testing	Explains test purpose and results in accessible terms	(39)
Treatment education	Surgery, radiotherapy, chemotherapy	Clarifies treatment pathways and expectations without decision-making	(22)
Symptom interpretation	Early neurological symptoms	Reinforces urgency and appropriate care-seeking behavior	(24, 48)
Health literacy support	Education outside clinic visits	Provides on-demand, personalized explanations	(45)

Given the importance of LLMs’ potential and the complexity of human behavior, their evaluation should extend beyond text similarity and performance to include human behavior. When assessing LLM-generated content for neuro-oncology patient education, structured quality metrics are essential. Previous studies in neuro-oncology have used expert-rated measures of accuracy, completeness, conciseness, and safety, along with objective metrics such as cosine similarity and readability (47). These provide a foundation for safety and informational quality. However, patient education also needs to consider readability, clarity, empathy, cultural sensitivity, uncertainty, and shared decision-making, especially since patients may face cognitive issues, distress, and low health literacy. Evaluation should include patient-centered outcomes such as comprehension, anxiety, trust, and behavior. While correctness, completeness, conciseness, and harm metrics are important, they are not enough. A comprehensive framework its essential to include clinical accuracy, readability, emotional tone, transparency, patient understanding, and usability, ensuring a multidimensional approach that aligns better with patient education goals.

The principal educational applications of LLMs in each brain tumor subtype are summarized in **Table 2**.

**Table 2:** LLMs performance across brain tumor subtypes.

Tumor subtype	Empirical evaluation status	Context of evaluation	Key findings	Evidence gaps
Meningioma	Extensively evaluated	Post-radiotherapy patient education	LLM-generated content rated as clear and relevant by >90% of patients; clinicians largely agreed on informational accuracy. Patients reported that earlier access to such explanations would have improved understanding and preparedness.	Long-term impact on comprehension, anxiety, and shared decision-making not yet assessed.
Pituitary Adenoma	Extensively evaluated	Patient question–answer scenarios	>82% of responses rated as trustworthy for general patient-level inquiries. Performance declined significantly for specialized or professional-level clinical questions.	Endocrine-specific nuance and the complexity of multidisciplinary management require further validation.
Glioblastoma	Evaluated (diagnostic context)	MRI interpretation and diagnostic reasoning	Advanced models frequently failed to identify subtle imaging features; some suggested alternative diagnoses (e.g., demyelination, edema). Highlights limitations in complex neuroimaging reasoning.	Real-world performance in patient education settings remains underexplored.
Gliomas(General)	Evaluated on decision-support context	Adjuvant therapy planning	Moderate-to-high expert consensus on LLM-suggested treatment plans despite low agreement on diagnostic interpretation. Suggests utility in summarizing established pathways rather than independent decision-making.	Limited to simulated settings; lacks prospective validation in clinical workflows.
Brain Metastases	Currently underexplored	General neuro-oncology education	Recognized as a clinically distinct category requiring tailored education; however, specific empirical LLM performance data were not synthesized in current literature.	Subtype-specific evaluation is urgently needed, particularly given diverse primary tumor origins and systemic therapy implications.
Pediatric Brain Tumors	Underexplored	—	Included in literature search scope, but no empirical studies directly assessing LLM performance in pediatric neuro-oncology education were identified.	Ethical, developmental, and caregiver-mediated education dynamics require focused research.
Vestibular Schwannoma and Hemangioblastoma	Underexplored	—	Referenced in search strategy but not represented in empirical performance studies within current literature.	Rare tumor subtypes remain largely unexamined in LLM-based patient education research.

### 3.4 Advantages of LLM-based educational tools

LLMs offer benefits as supplemental educational tools in patient-centered care, particularly for complex conditions such as brain tumors. First, they help bridge health literacy gaps by translating intricate medical information from extensive medical texts into accessible language to improve understanding and engagement (50). Second, LLMs provide continuous access to educational resources outside of visits, allowing patients to ask questions anytime and better prepare for appointments. Third, they also customize explanations to suit individual needs, adjusting tone and complexity to improve understanding and encourage patient participation in decision-making (45). Eventually, LLMs can enhance the readability of patient materials through tailored prompts and refinements. Although they enhance accessibility, personalization, and support, professional oversight remains crucial to ensure safety and relevance.

### 3.5 Risks, limitations, and ethical considerations

LLMs, while promising as educational tools, pose significant risks and ethical issues in neuro-oncologic patient education. One key issue is hallucination, where LLMs may produce plausible yet incorrect or incomplete medical information (51). In brain tumor care, where discussions often involve prognosis, treatment risks, and imaging, such inaccuracies can cause confusion, false reassurance, or unnecessary worry. Recent approaches have incorporated grounding strategies such as retrieval-augmented generation (RAG), which constrain outputs to curated knowledge sources, thereby minimizing the risk of hallucinations and improving factual alignment in patient-related explanations (52). Another concern is overtrust and automation bias. Because LLMs generate fluent, authoritative responses, patients might view AI-generated information as clinically valid, even if it lacks nuance or context (53, 54). This is especially problematic in neuro-oncology, where patients often face emotional vulnerability, cognitive issues, or decision-making stress. Relying uncritically on LLMs could hinder rather than support shared decision-making.

LLMs’ output quality varies and adds complexity, as performance depends on prompt design, model version, and input quality, leading to inconsistent completeness and sometimes missing relevant clinical details. Such variability is risky for topics such as treatment side effects, functional outcomes, or follow-up care, where partial information can distort expectations (45).

Beyond technical performance, extended interaction with conversational AI may lead to complex emotional bonds between patients and LLM-based tools. Research on human–AI interaction and mental health chatbots indicates that users may develop emotional dependence, heightened expectations of empathy, and disappointment when AI responses do not meet their support needs (55, 56). In vulnerable groups, like patients with brain tumors experiencing anxiety, cognitive issues, and emotional distress, these effects could be stronger. Nonetheless, the long-term emotional, psychological, and behavioral impacts of ongoing patient–LLM interactions in neuro-oncology remain poorly understood. Longitudinal and qualitative research will be vital to determine whether such interactions improve coping and engagement or pose risks, such as overreliance, emotional substitution, or unmet support expectations.

Ethical concerns also involve data privacy and confidentiality. Patient-facing LLM tools may handle sensitive health data outside regulated clinical settings, raising issues of data security, informed consent, and secondary use. Moreover, biases in training data can lead to unfair or culturally insensitive explanations, worsening disparities in health literacy and access.

Historically, LLMs in healthcare have been considered a problem because they are not licensed medical professionals. However, the approach to accountability is evolving from uncertainty to structured regulation. Recently, LLMs have shifted from experimental tools to regulated medical devices. A notable example is the Prof. Valmed system, which in late 2024 became one of the first LLM-powered clinical decision-support tools to obtain a Class IIb CE mark under the EU Medical Device Regulation (MDR) (57). This high-risk classification demands a thorough quality management system and clinical evidence, placing legal responsibility for performance on the manufacturer, while allowing clinicians to remain the final decision-makers. Similarly, the FDA addresses the changing nature of LLMs with the Predetermined Change Control Plan (PCCP) framework, enabling manufacturers to predefine updates and fine-tune procedures post-market to ensure safety standards are maintained (58). Moreover, the accountability gap is being narrowed through Human-in-the-Loop architectures mandated by the EU AI Act. These frameworks deploy LLMs as assistance rather than autonomous agents. For example, integrating GPT-4 into clinical workflows for drafting patient responses and triage involves a review-and-release process. The LLM provides evidence-based suggestions using RAG, but a licensed professional must validate the output before it reaches the patient. In structured deployments, RAG can be configured to retrieve information from curated institutional knowledge bases or hospital-approved data sources, and the clinician verifies this information to ensure accountability and reduce the risk of misinformation and hallucinations, thereby minimizing potential patient harm (52).

Locally hosted RAG architectures should be seen as risk-mitigation tools rather than complete solutions. Limiting retrieval to institutional databases may improve data governance and reduce external data sharing, but it does not eliminate biases in pretrained models, prevent automation bias, or prevent patient misinterpretation. Ensuring cultural sensitivity, fairness, and contextual relevance requires ongoing human oversight, multidisciplinary review, and regular auditing. Thus, accountability in LLM-assisted patient education is best understood as a layered approach that combines regulatory compliance, technical safeguards like RAG, and clinician-led validation processes.

In addition to architectural safeguards such as RAG and human-in-the-loop validation, domain-specific fine-tuning represents another potential strategy to improve performance and reduce bias. Fine-tuning LLMs on curated neuro-oncology educational materials, institutional guidelines, and plain-language patient resources may enhance factual alignment and readability while reducing jargon density. Reinforcement learning with clinician feedback could further refine safety and tone. However, fine-tuning does not eliminate structural biases embedded in pretraining data nor fully prevent hallucinations or overgeneralization (59). Therefore, it should be viewed as a complementary mitigation strategy within a broader governance, monitoring, and clinician oversight framework rather than a standalone solution.

### 3.6 Framework for responsible integration of large language models in clinical practice

To safely incorporate LLMs into neuro-oncology patient education, a structured, clinically guided integration framework is crucial. This approach relies on a human-in-the-loop model, in which LLMs serve only as educational assistants rather than as autonomous clinical decision-makers (**Table 3**). Clinician oversight is vital for contextualizing AI outputs, verifying accuracy, and maintaining professional responsibility (60, 61). First, the intended use must be clearly defined, limiting LLMs to explaining diagnoses, imaging results, treatments, and general prognosis, while avoiding diagnostic decisions or treatment advice. In addition, clear boundaries help prevent overreliance and automation biases, especially for vulnerable neuro-oncology patients.

**Table 3:** Risks, limitations, and ethical challenges of LLM use in neuro-oncology.

Category	LLM issue	Potential impact	Mitigation strategy	Key references
Hallucinations	Incorrect or incomplete information	False reassurance or anxiety	Clinician review and uncertainty disclosure	(51)
Overtrust	AI perceived as clinical authority	Impaired shared decision-making	Explicit educational-only labeling	(53, 54)
Output variability	Sensitivity to prompts and inputs	Inconsistent information quality	Standardized prompting	(41, 45)
Readability	High literacy level of outputs	Reduced patient comprehension	Prompt-guided simplification	(7, 59)
Data privacy	Handling of sensitive data	Confidentiality risks	Secure clinical platforms	(48)
Bias and equity	Cultural or systemic bias	Worsened disparities	Oversight and adaptive content	(60)
Accountability	Unclear responsibility	Ethical and legal ambiguity	Human-in-the-loop governance	(60–62)

Second, prompt-and-output governance helps ensure consistent, patient-centered communication. In clinical practice, a structured prompt may take the form of a template-based instruction embedded within the electronic health record (EHR), specifying target readability (e.g., ≤8th-grade level), mandatory uncertainty disclosure statements clarifying that the output does not constitute medical advice, scope limitations, and requirements that all medical terminology be defined. Automated readability scoring can be applied post-generation, with iterative simplification triggered when predefined thresholds are exceeded. These safeguards address the tendency of LLMs trained predominantly on scientific literature to generate linguistically dense outputs rich in medical jargon.

Uncertainty disclosures may be implemented through predefined, automatically appended institutional statements that clarify scope, reinforce clinician authority, and limit overreliance. In addition, outputs may include references to institutional or guideline-based sources retrieved through a hospital-based RAG system, along with structured confidence indicators. This approach encourages patients to interpret responses as supportive educational information rather than definitive medical guidance.

Clinician oversight involves a validation workflow in which responses are reviewed, modified, and approved by a licensed professional before being shared with the patient. All outputs should be logged within the clinical system to enable auditing, quality review, and monitoring of hallucination rates or policy deviations. Integration into patient portals should occur within secure clinical environments rather than isolated external platforms and should incorporate real-time monitoring dashboards and escalation pathways for clinician review of high-risk outputs. Prior to deployment, predefined safety metrics, including acceptable accuracy thresholds, hallucination rate limits, readability targets, and documentation standards, should be established. Finally, AI literacy training for clinicians and patients is essential to ensure that LLM outputs are viewed as educational tools rather than substitutes for medical judgment. Together, these measures translate governance principles into actionable safeguards while preserving clinician accountability and patient safety (62). At the same time, periodic reviews by institutional AI governance committees can help ensure compliance and quality control.

Regarding legal responsibility, it may be wise to divide it into three domains: Legal responsibility in LLM-assisted patient education could involve shared accountability among manufacturers (for system performance), healthcare institutions (for implementation governance), and clinicians (for final decision validation) (63).

### 3.7 Future directions

While LLMs show promise in neuro-oncology, their use remains inconsistent across tumor types and settings. Future research should focus on outcome-based studies that address the clinical features of brain tumors. Validation must be tailored to subtypes, especially aggressive cancers like glioblastoma, brain metastases, pediatric tumors, and less common tumors like hemangioblastomas, which are still understudied. Research should also evaluate real-world patient outcomes, including understanding, decision-making, anxiety, treatment adherence, and the psychological effects of human–AI interaction. Improving health literacy remains essential and involves adaptive readability, multilingual options, and culturally appropriate communication to reduce disparities. In addition, developing multimodal LLMs that interpret imaging data alongside reports can enhance the interpretability of neuroimaging. Integration should occur within secure electronic health records and patient portals, with clinician oversight, validation, and audit systems for accountability. Ultimately, safe deployment depends on alignment with regulations such as the EU AI Act and the FDA’s PCCP. Establishing validation, governance, and accountability standards requires ongoing collaboration among clinicians, informaticians, ethicists, and policymakers as LLM technology advances.

## 4 Conclusions

In summary, providing effective patient education in neuro-oncology is an ongoing challenge due to the complexity of brain tumors, the importance of imaging, and the cognitive and emotional vulnerability of patients and their families. LLMs show promising potential to support patient-centered communication by simplifying complex neuro-oncologic information into more accessible, personalized explanations and by enhancing clinician guidance beyond the limited time available during clinical visits. However, current evidence also reveals significant limitations, including hallucinations, variability, overtrust, and unresolved ethical and accountability issues, which preclude their use as standalone decision-making tools in clinical practice. When used responsibly with clinician oversight, LLMs can act as educational aids to improve understanding, engagement, and health literacy without replacing professional judgment. Future success will require thorough evaluation, structured governance, and adherence to ethical and regulatory standards. If implemented carefully, LLMs could help create a more informed, supported, and empowered experience for patients facing the challenging journey of brain tumor diagnosis and treatment.
